# Development and Validation of an Ultrasonography-Based Machine Learning Model for Predicting Outcomes of Bruxism Treatments

**DOI:** 10.3390/diagnostics14111158

**Published:** 2024-05-31

**Authors:** Kaan Orhan, Gokhan Yazici, Merve Önder, Cengiz Evli, Melek Volkan-Yazici, Mehmet Eray Kolsuz, Nilsun Bağış, Nihan Kafa, Fehmi Gönüldaş

**Affiliations:** 1 Department of Dentomaxillofacial Radiology, Faculty of Dentistry, Ankara University, Ankara 06560, Turkey; call53@yahoo.com (K.O.); m_eraykolsuz@yahoo.com (M.E.K.); 2 Department of Dental and Maxillofacial Radiodiagnostics, Medical University of Lublin, 20-059 Lublin, Poland; 3 Medical Design Application and Research Center (MEDITAM), Ankara University, Ankara 06000, Turkey; 4 Department of Oral Diagnostics, Faculty of Dendistry, Semmelweis University, 1088 Budapest, Hungary; 5 Department of Physiotherapy and Rehabilitation, Faculty of Health Sciences, Gazi University, Ankara 06490, Turkey; gokhanyazici38@hotmail.com (G.Y.); nkaratas@gazi.edu.tr (N.K.); 6 Department of Oral and Maxillofacial Radiology, Faculty of Dentistry, Ankara University, Ankara 06560, Turkey; merveonder_16@hotmail.com (M.Ö.); dt.cengizevli@gmail.com (C.E.); 7 Department of Physiotherapy and Rehabilitation, Faculty of Health Sciences, Yuksek Ihtisas University, Ankara 06520, Turkey; melek_volkan89@hotmail.com; 8 Department of Periodontology, Faculty of Dentistry, Ankara University, Ankara 06560, Turkey; nilsunbagis@yahoo.com; 9 Department of Prosthetic Dentistry, Faculty of Dentistry, Ankara University, Ankara 06500, Turkey

**Keywords:** artificial intelligence, bruxism, machine learning, ultrasound

## Abstract

Background and Objectives: We aimed to develop a predictive model for the outcome of bruxism treatments using ultrasonography (USG)-based machine learning (ML) techniques. This study is a quantitative research study (predictive modeling study) in which different treatment methods applied to bruxism patients are evaluated through artificial intelligence. Materials and Methods: The study population comprised 102 participants with bruxism in three treatment groups: Manual therapy, Manual therapy and Kinesio Tape or Botulinum Toxin-A injection. USG imaging was performed on the masseter muscle to calculate muscle thickness, and pain thresholds were evaluated using an algometer. A radiomics platform was utilized to handle imaging and clinical data, as well as to perform a subsequent radiomics statistical analysis. Results: The area under the curve (AUC) values of all machine learning methods ranged from 0.772 to 0.986 for the training data and from 0.394 to 0.848 for the test data. The Support Vector Machine (SVM) led to excellent discrimination between bruxism and normal patients from USG images. Radiomics characteristics in pre-treatment ultrasound scans of patients, showing coarse and nonuniform muscles, were associated with a greater chance of less effective pain reduction outcomes. Conclusions: This study has introduced a machine learning model using SVM analysis on ultrasound (USG) images for bruxism patients, which can detect masseter muscle changes on USG. Support Vector Machine regression analysis showed the combined ML models can also predict the outcome of the pain reduction.

## 1. Introduction

Bruxism, which is defined as “repetitive jaw muscular activity characterized by the clenching or grinding of teeth and/or bracing or thrusting of the mandible,” leads to changes in masseter muscle morphology [1]. These changes include inflammation and hypertrophy of the muscles, chronic local muscular contracture, and myofascial pain. Despite recent advances in the treatment and rehabilitation of bruxism and masseter muscle diseases, the question of an effective treatment or rehabilitation approach is still unanswered. Medical professionals are engaged in finding novel treatments, rehabilitation protocols, and techniques that could optimize rehabilitation time and advance healing. One of the most important underlying principles in a successful treatment is performing correct medical diagnosis and assessment [2,3,4]. Ultrasonography (USG) is frequently used in the medical imaging of bruxism [5]. USG has several advantages: it is portable, does not use ionizing radiation, is cost-effective, and provides instantaneous imaging [6].

The field of medical imaging, including USG, has experienced significant advancements in recent years via the utilization of artificial intelligence (AI). This has facilitated the creation of an image analysis methodology that is data-driven and relies on models that are less biased compared to traditional approaches, which heavily rely on heuristic assumptions regarding the appearance of objects. Machine learning (ML) is a subdomain of AI wherein machines acquire the ability to do a particular job by focusing on the identification of patterns within existing data through mathematical analysis of accessible datasets via the utilization of algorithms, rather than relying on explicit programming with predetermined instructions [7,8]. These algorithms subsequently undergo self-training processes to learn from the data and provide predictions based on newly acquired information [9,10]. This has resulted in improved cost-effectiveness, time efficiency, less reliance on human expertise, and a reduction in medical errors [4,11,12,13] and has provided assistance in making precise diagnoses [14,15,16].

Radiomics is an emerging field that focuses on the creation of relevant statistical models using a substantial quantity of high-dimensional extractable features derived from medical imaging data. These features may be used in conjunction with genomic or clinical data to enhance diagnostic, prognostic, and treatment monitoring processes [17]. In radiomics, a statistical model is constructed using ML algorithms based on specific biological or clinical inquiries and any existing prior knowledge [18,19]. Radiomics platforms have the potential to discover unique imaging algorithms for quantifying radiographic features including intensity, shape, and texture that enable prognostic, predictive imaging modalities to be built [19]. Moreover, log-linear regression (LOG) features can also be used for generating AI models using radiomics to improve predicted outcomes from a linear regression model [20].

USG-based machine learning techniques have gained significant importance in medical diagnostics. However, investigations of USG-based machine learning models used in the prediction of bruxism treatments are scanty and have not generated a validated and established prediction model [4]. 

Therefore, this study aimed to (1) develop a USG-based ML model to distinguish the masseter muscles in patients with bruxism compared to normal subjects and (2) predict the treatment outcome for pain reduction among bruxism patients from various treatment modalities, with validation of the predictive accuracy and clinical efficacy of learning model based on pre- and post-treatment USG images and clinical evaluations. 

This study has two hypotheses; the first hypothesis of this study (H1) is that USG-based ML can distinguish the masseter muscles in patients with bruxism compared to normal subjects. The second hypothesis (H2) is that the treatment outcome for pain reduction among bruxism patients can be predicted via USG-based ML from various treatment modalities.

The primary hypothesis herein was that balance and functional mobility would improve following PFEs in patients with SCP. 

## 2. Materials and Methods

The Research Ethics Committee of Gazi University (Reference no: 2022-321) approved this retrospective analysis of anonymous data and waived the requirement for informed consent. The protocol for the study was in accordance with the guidelines of the Declaration of Helsinki. 

The retrospective data of patients were included if they were older than 18 years of age and had been diagnosed with bruxism. Exclusion criteria were the absence of more than two molar teeth; treatment with removable partial dentures; neurological, psychiatric, or systemic diseases; alcohol and/or drug addiction; ongoing dental or physical therapy; the use of an occlusal splint during assessment; pregnancy; active cancer; or planned surgical procedures. Patients were also excluded if they had problems with their molar teeth, such as pericoronitis or supraeruption, and if they had been treated for temporomandibular disorders in the previous three months. 

The study population consisted of 102 participants who were admitted to Ankara University Faculty of Dentistry with reported teeth grinding during sleep. Bruxism was diagnosed through self-report and clinical examination. In addition to the patients’ self-reports, dental wear consistent with attrition and clinical signs and symptoms such as jaw muscle discomfort, fatigue, and/or pain upon palpation were evaluated. Neither polysomnography nor electromyography were used. The Fonseca Anamnestic Index was employed for self-reporting as employed by Cavallo et al. [21] for the diagnosis of bruxism; it can also be used to diagnose temporomandibular joint dysfunction [22]. The index can provide relevant information in a short period and can be understood easily, allowing patients to be categorized according to the severity of bruxism and/or temporomandibular joint disorder [21,22,23,24,25,26].

Out of 102 participants who applied to Ankara University Faculty of Dentistry, 78 participants were diagnosed with bruxism. When the remaining 24 participants were examined, it was determined that they did not actually have bruxism. Therefore, they were included in the control group. 

### 2.1. Outcome Variables

**Figure 1:**
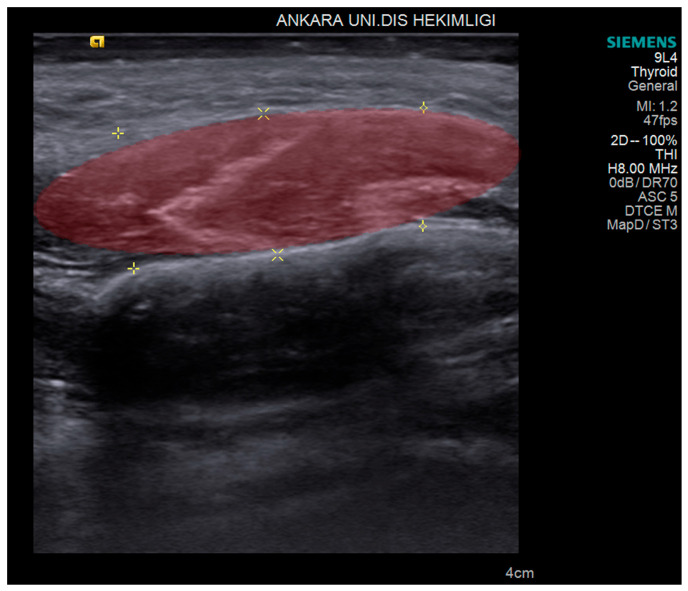
Volume of interest (red area) outlined on the ultrasonographic image of the masseter muscle that was used for machine learning and radiomics analysis. Yellow markers indicate the edge of masseter muscle.

The primary outcome was to distinguish the masseter muscles of the bruxism patients from control subjects by generating an ML algorithm. The secondary outcome was to predict treatment outcomes for pain reduction among bruxism patients from various treatment modalities that were derived from pre- and post-treatment USG images and clinical evaluations. The USG image of masseter muscle thickness measurements before and after treatment are shown in Figure 1.

### 2.2. USG Imaging

Two USG consoles, the ProSound Alpha 6 (Hitachi Aloka Medical Ltd., Tokyo, Japan) with a linear probe 5–13.3 MHz (UST-5413, Hitachi Aloka Medical Ltd., Tokyo, Japan) and the high-resolution ACUSON S 2000 ultrasound machine (Siemens, Munich, Germany) with a 4–9 MHz linear probe (9L4 Transducer), were used in this study. Extraoral scanning was performed with different frequency bandwidths. After adjusting the probe to the desired trajectory, images were obtained using both probes. Two ultrasonographers (C.E., K.O.) performed all USG scanning and interpretations. Both ultrasonographers were dentomaxillofacial radiologists with 10 and 5 years of experience in acquiring and interpreting USG. They were blinded to the bruxism or control status of the participants being examined.

During the USG procedure, the patient’s head was adjusted to the measured side. Imaging was performed by holding the probe perpendicular to the masseter muscles close to the level of the occlusal plane, approximately in the middle of the mediolateral distance of the ramus, taking care to keep the probe perpendicular to the ramus. The probe was moved in the anterior–posterior direction to produce transverse sections. The operators were free to position the probe when acquiring the images.

As USG scanning is a dynamic modality, we acquired USG images consecutively frame by frame to have as many images as possible from the anatomical region of both left and right masseter muscles, to be used later for both diagnosing and labeling. In total we obtained 623 USG images from 102 patients. 

The entire masseter muscle image set was stored in the USG device for further analysis (Figure 1). The ultrasonographers each conducted 2 separate USG sessions independently for all participants, at baseline and following 4 weeks of treatment for bruxism patients and at the same times for the control group. 

### 2.3. USG Image Segmentation

The thickness of the masseter muscles was measured on the stored USG images by a single radiologist (C.E.) who was blinded to the clinical status of the patients. Volumes of interest (VOIs) were delineated manually by the radiologist (Figure 1). All contours were then reviewed by the senior radiologist (K.O). If a discrepancy in volume measurement exceeded 5%, the senior radiologist decided on the contours and border of the masseter muscles. 

All 623 images were preprocessed to make them suitable for the AI model. Preprocessing included resizing, cropping, padding, rotating, and augmenting the images to make them uniform, as well as reduction in noise and variation and the avoidance of overfitting. We created a standardization protocol by transforming the pixel values to make them compatible with the input range and distribution of the AI model. After this step-in total, 235 VOIs were delineated from 623 USG images for model creation.

### 2.4. Treatment Protocols of Patients

Patients with bruxism were treated in one of three protocols. (1) Manual therapy (MT) consisted of kneading, friction, intramuscular stretching, sliding, and ischemic compression on trigger points, applied on the masseter and temporalis muscles intra- and extraorally. Stretching exercises and cervical mobilization techniques were also performed. (2) In some patients, application of Kinesio Tape was combined with manual therapy (KTMT), in which web-cut strips of epidermis-dermis-fascia (EDF) KT were crossed over the masseter muscle area with 0–5% tension in the maximum stretched epidermis position. Kinesio Tape was applied immediately after MT and removed one day before the physiotherapy session, leaving it in place for at least 2 days [27]. In the MT and KTMT treatments, each session lasted 30 min and was performed by a physiotherapist who had a Master Certification in manual therapy. (3) In the third protocol, Botulinum Toxin-A (BT-A) was injected into the masseter muscles for pain reduction and reduction in bruxism effects. All patients were informed of the possible side effects of the BT-A injection, and written signed consent forms were collected from them. For a dose of 1.0 U/0.1 mL, 100 U frozen dried BT-A (Botox, Allergan, Inc., Irvine, CA, USA) was diluted with 2 cc saline, and 30-gauge injectors were used for all the injections. The patients were positioned horizontally according to the Frankfurt horizontal plane. All injections were performed by the same clinician (N.B.). A dose of 25 U for each masseter muscle (total of 50 U for each patient) was injected. Only one session of application was performed. The 78 bruxism patients were classified into these 3 treatment subgroups: 20 in the MT subgroup, 19 in the KTMT subgroup, and 39 in the BT-A subgroup. 

**Figure 2:**
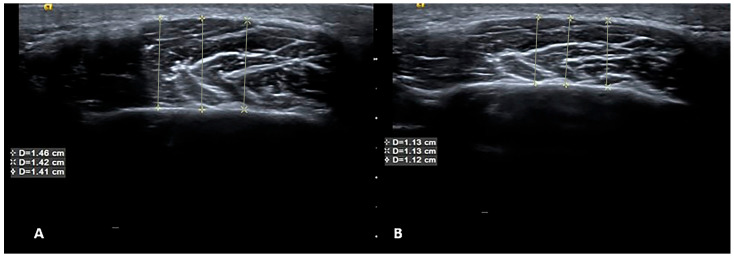
USG at baseline and 4 weeks after BT-A therapy. Ultrasonographic images of the masseter muscle before (**A**) and after (**B**) botulinum toxin injection. As seen from measurements listed in the text boxes superimposed on the images, muscle volume decreased after treatment, which indicates that the treatment was successful. Yellow Lines indicates the measurement of the muscles from different region.

Age, sex, and body mass index were recorded for all participants in the patient and control groups. Maximum mouth opening, pain (visual analog score), pain on palpation, and sleep quality index (using the Pittsburg quality index) [28] were also assessed with scales regarding the associated symptoms. Pain thresholds were evaluated using an algometer (Lafayette Model 01165A, Lafayette Instrument Company, Inc., Lafayette, IN, USA). Evaluations of all participants were performed at baseline and following 4 weeks of different physiotherapy applications and at the same times for the control group. USG images and clinical information were included in the analysis. An example of USG at baseline and 4 weeks after BT-A therapy is presented in Figure 2.

### 2.5. Radiomics Evaluation

A radiomics platform (Huiying Medical Technology Co., Ltd., Beijing, China, http://en.huiyihuiying.com/, accessed on 23 April 2024) was used to manage imaging data, clinical data, and subsequent radiomics statistical analysis. This platform is capable of extracting radiomics features from 2D and 3D images and binary masks on different imaging modalities. In this research, the training dataset and test dataset were separated by the random method with ratio 8:2, with 782 randomly sampled seeds.

### 2.6. Feature Extraction

In total, 2818 quantitative imaging features were extracted from USG images with the Radcloud platform (http://radcloud.cn/, accessed on 23 April 2024) in 3 groups. Group 1 (first-order statistics) comprised descriptors that quantitatively describe the distribution of voxel intensities in the image using basic and commonly used metrics. Group 2 (shape- and size-based features) included three-dimensional characteristics reflecting the shape and size of the region. Group 3 (texture features) were calculated from gray level co-occurrence and gray level run-length texture matrices, which can quantify regional heterogeneity differences. Texture features containing gray level co-occurrence matrix (GLCM) and gray level size zone matrix (GLSZM) were used. Intensity and texture features can be calculated on the original image and derived images, obtained by applying several filters such as exponential, logarithm, square, square root, and wavelet (wavelet-LHL, wavelet-L, wave-let-HLL, wavelet-LLH, wavelet-HLH, wavelet- HHH, wavelet-HHL, wavelet-LLL). After image segmentation, the identified radiomics features were calculated and associated with features for masseter muscle identification and bruxism treatment prediction from USG images (Table 1). The features complied with definitions established by the Imaging Biomarker Standardization Initiative (IBSI) [29].

For feature extraction, dimensionality reduction and selection of task-specific features for best performance were performed. To reduce the redundant features, the feature selection methods included the variance threshold. SelectKBest and the least absolute shrinkage and selection operator (LASSO) were used for this purpose. With the variance threshold set at 0.8, eigenvalues of the variance smaller than 0.8 were removed. The SelectKBest method, which belongs to a single variable feature selection method, used the *p*-value to analyze the relationship between the features and the classification results; all features with *p*-values smaller than 0.05 were used. For the LASSO model, ‘L1 regularize’ was used as the cost function. The error value of the cross test was 5, and the maximum number of iterations was 1000.

### 2.7. Consensus Clustering 

A consensus clustering was also used for clustering the radiomics features after extraction from the training sets. In this study, the range of a suitable amount of clusters was estimated. According to this range, the amount of clusters that produced the highest median cluster consensus compared to the other clusters was selected. The definition of cluster consensus was the average consensus among feature pairs that belong to the identical cluster. 

Subsequent to clustering, integration of the chosen features with clinical variables was performed in order to construct a primary dataset with good stability and reliability. With regard to the other significant variables, the *p*-value threshold was fixed at 0.05 [30]. During the preliminary filtering stage, univariate analysis was employed for the purpose of filtering out multiple unconnected features, which allowed the optimal feature established via univariate analysis to be calculated. 

### 2.8. Statistical and Machine Learning Analysis

Computer-generated random numbers were used to assign 80% of the VOIs to the training dataset and 20% of VOIs to the test dataset. Based on the selected features, there are several supervised learning classifiers available for classification analysis, which creates models that attempt to separate or predict the data with respect to an outcome or phenotype (for instance, patient outcome or response). In this study, the radiomics-based models were constructed with 6 classifiers: k-Nearest Neighbor (KNN), Support Vector Machine (SVM), eXtreme Gradient Boosting (XGBoost), Random Forest (RF), Logistic Regression (LR), and Decision tree (DT). The test method was used to improve the effectiveness of the model. The best learning classifier was selected for testing the secondary outcome, defined as clinically reduced pain and bruxism conditions after treatment.

In order to calculate outcome measures, the ML classifiers must indicate if the USG image has texture features representing bruxism or control. The outcomes’ measures were based on the interpretation of the texture features as disease or control compared to the actual known status of the patient. To assess the predictive performance, the area under curve (AUC) generated through receiver operating characteristic (ROC) analysis was used both in training and test datasets. The scale that was used to interpret the ability of the classifier to discriminate between two conditions designated AUC values of 0.5–0.7 as poor discrimination, 0.7–0.8 as acceptable discrimination, 0.8–0.9 as excellent discrimination, and >0.9 as outstanding discrimination [30]. Sensitivity and specificity values were also calculated. In addition, four other indicators were calculated to evaluate the performance of the classifiers in this study: precision (P) = true positives/(true positives + false positives), recall (R) = true positives/(true positives + false negatives), F1 = 2 * (precision * recall)/(precision + recall), and support, which was defined as the number of samples of the true response that lies in each class of target values.

Statistical differences between the predictive radiomics features of the models were tested using the Mann–Whitney U test in relation to outcomes for pain reduction. Statistical analyses were performed using IBM SPSS Statistics package 21.0 (IBM Corp. Released 2012. IBM SPSS Statistics for Windows, Version 21.0. Armonk, NY, USA: IBM Corp). A *p*-value was considered to be statistically significant if less than 0.05.

## 3. Results

The mean age of the 78 bruxism patients (35 men and 43 women) was 52 years ±13.86 with an age range of 20–81 years. The mean age of the 24 control subjects (14 women and 10 men) was 48 ± 12.36 with a range of 19–74 years. There was no statistically significant difference in the groups according to age or sex (*p* > 0.005).

### 3.1. Machine Learning and Radiomics Results

After dimensionality reduction and the selection of task-specific features, a total of 922 of the 2818 extracted features were selected using the variance threshold method. Following the application of the SelectKBest method, we selected 132 features, and finally, after application of the LASSO algorithm, we selected 6 optimal features for ML:

- Ibp-2D_firstorder_variance;
- Exponential_glszm_ZoneVariance;
- Wavelet-LLL_firstorder_variance;
- Wavelet_HLH_glszm_SmallAreaHighGrayLevelEmphasis;
- Wavelet_LLH_glszm_SmalldepedneceHighGrayLevelEmphasis;
- Ibp-2D_firstorder_10percentile.

These features were categorized into three groups. Group 1 (first order statistics) consisted of descriptors that quantitatively delineate the distribution of voxel intensities with in the image through commonly used and basic metrics. Group 2 (shape- and size-based features) contained three-dimensional features that reflect the shape and size of the region. Group 3 (texture features) was calculated from gray level co-occurrence and gray level run-length texture matrices, which can quantify region heterogeneity differences.

### 3.2. Diagnostic Performance of Machine Learning Using Six Supervised Learning Classifiers

**Figure 3:**
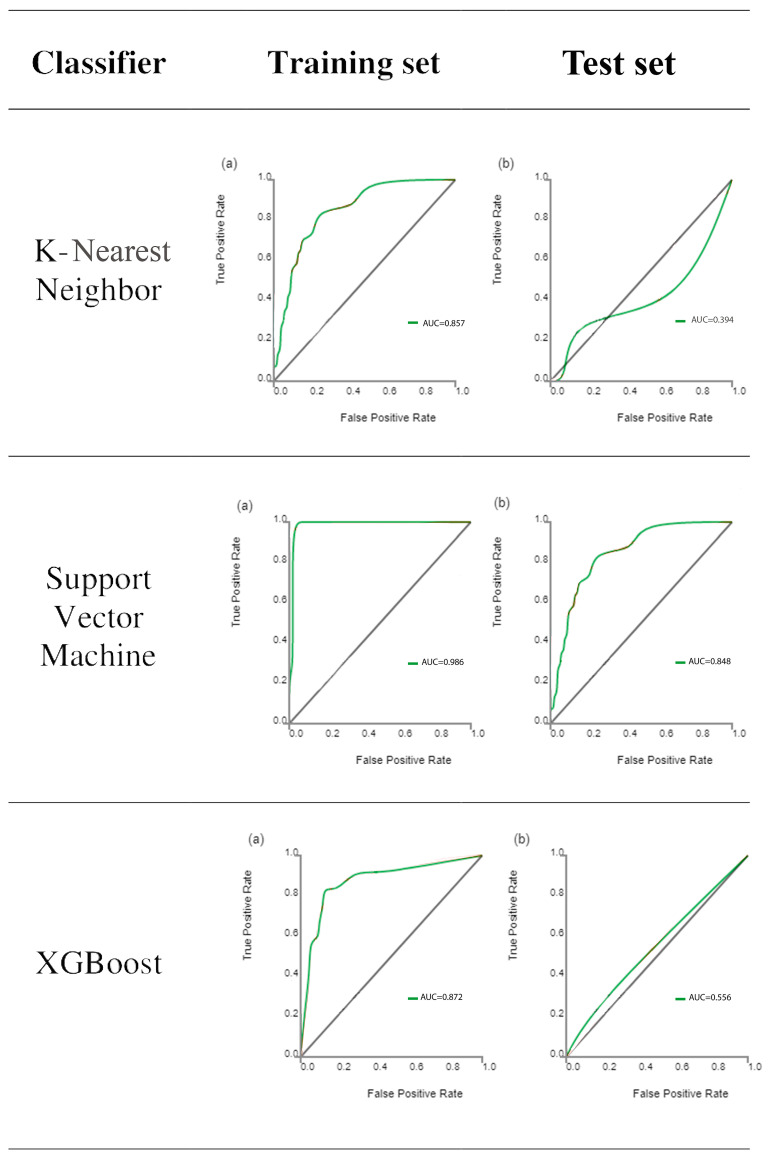

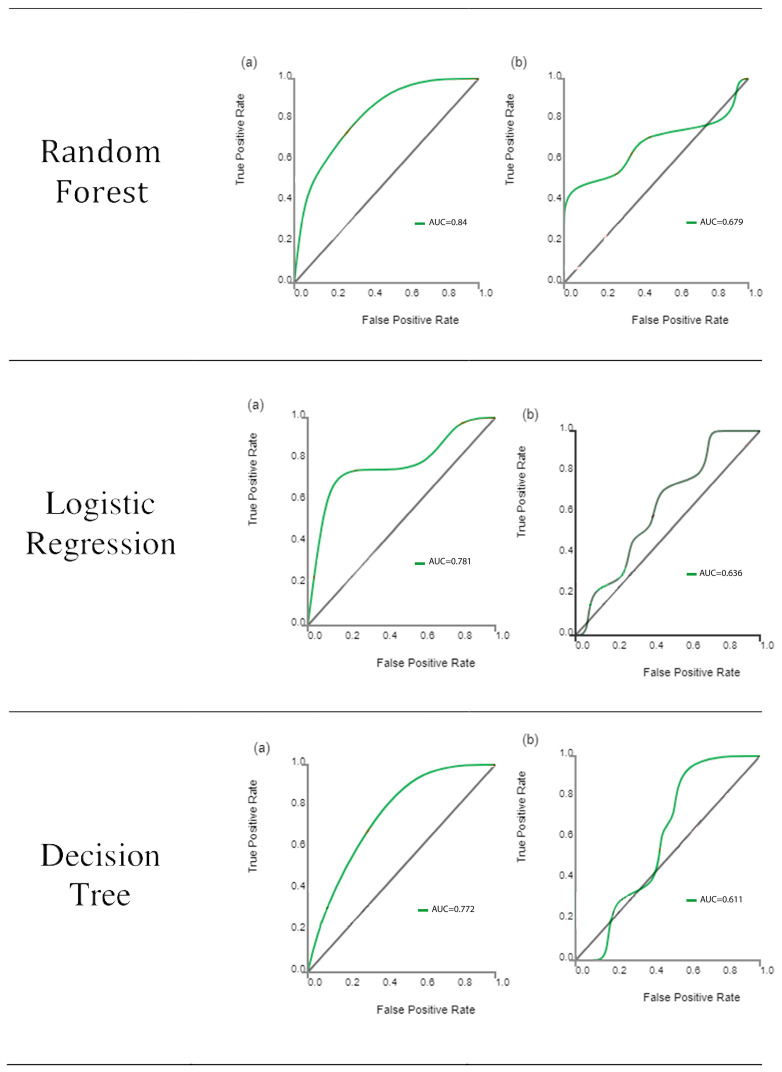
Receiver operating characteristic curve analysis results of the ability of the six classifiers to differentiate between bruxism patients and normal subjects for the training set (**a**) and test set (**b**) datasets. The diagonal line represents random chance.

Following feature extraction, ML classifiers were tested. The ROC curves of learning classifiers are presented in Figure 3 for the training and test datasets, representing the ability of the datasets to differentiate between the masseter muscles of bruxism patients and control subjects. The AUC values of all machine learning methods ranged from 0.772 to 0.986 for the training data (Table 2) and from 0.394 to 0.848 for the test data (Table 3). SVM exhibited the greatest discriminating ability among all ML classifiers in both datasets, with AUC of 0.986 in the training dataset indicating outstanding discrimination and AUC of 0.848 in the test dataset indicating excellent discrimination. The sum of sensitivity and specificity was 1.62 in the training set and 1.78 in the test set. It has been stated that this sum must be at least 1.5 for the test to have any meaning in predicting the presence or absence of disease [31]. On the other hand, KNN is completely valueless for the test dataset, with AUC = 0.394.

The diagnostic performance according to the four indicators are listed for training (Table 4) and test (Table 5) datasets. For the training set, the ranges for the control group were precision (0.80–0.91), recall (0.90–0.98), F1-score (0.86–0.95), and support (40), while the ranges for the bruxism group were precision (0.50–0.90), recall (0.14–0.64), F1-score (0.24–0.75), and support (12). For the test set, the ranges for the control group were precision (0.77–0.85), recall (0.91–1.0), F1-score (0.83–0.92), and support (11), while the ranges for the bruxism group were precision (0–1.0), recall (0–0.33), F1-score (0–0.50), and support (3). The highest scores were achieved with the SVM machine learning method for the test set (Figure 3).

The evaluation for the diagnostic performance data based on the discrimination between bruxism and control patients (Table 5) indicates that similar values were found both in the separate and cumulative data analysis.

### 3.3. Predictive Performance of Models for All Patients

Using both clinical and radiomics features, the best learning classifier (SVM) was selected for testing the secondary outcome of pain reduction in terms of clinical evaluation after treatment procedures. Performances of the clinical, radiomics, and combined models were evaluated based on SVM regression for the prediction of pain reduction and is summarized in Table 6. 

Combined features were selected for the prognostic of pain reduction following treatments regarding, respectively, the clinical, radiomic, and combined mode. In the clinical model, patients with poor outcomes for pain reduction were found in relation to age, BT-A injections alone. Radiomic features showed coarse and more non-homogeneous muscles after USG application in pre-treatment, which is more likely to lead to poor outcomes for pain reduction (Table 6). The reduction in pain on palpation is negatively correlated with SVM regression analysis, which is statistically significant before and after treatment. This can be clearly seen in Table 6.

## 4. Discussion

In this study, we aimed to develop a predictive model for the outcome of bruxism treatments using USG-based machine learning techniques. The findings of this study showed that the presence of bruxism can be detected via a USG-based machine learning technique. Furthermore, the outcome of pain reduction can also be predicted via combined ML models. After a series of steps involving dimensionality reduction and feature selection, a set of six optimal radiomics features were identified for the machine learning model. The selected features were derived from both first-order statistics and shape-based descriptors. The machine learning classifiers demonstrated reasonable performance in differentiating between bruxism patients’ masseter muscles and those of normal subjects, as indicated by the AUC values. However, it is important to note that the AUC values varied between the training and test datasets, indicating the need for further validation and improvement. Moreover, we evaluated the predictive performance of our models for all patients, including the secondary outcome of pain reduction. The best learning classifier (SVM) was selected for this evaluation, and the combined features (clinical and radiomics) were used. The results showed associations between poor outcomes for pain reduction and factors such as age and specific treatment procedures, as identified by the clinical and radiomic models. These findings highlight the potential of combining clinical and radiomic features in predicting treatment outcomes and assessing pain reduction in bruxism patients. 

Bruxism, which is very common in society, causes serious problems affecting the maxillofacial region such as maxillofacial pain, limitation in mouth opening, and decreased function in the temporomandibular joint. Since bruxism causes different problems in different regions, especially the results mentioned, bruxism patients should be approached in a multidisciplinary manner. Therefore, bruxism patients should be examined multidisciplinarily by psychiatrists, dentists, and physiotherapists [24,25]. In bruxism patients, the treatment is directed to the agent and the symptom. Bruxism can cause masticatory muscle hypertrophy, particularly in the masseter muscle. Manual therapy, Kinesio Tape, and BT-A are muscle-oriented treatments used in the treatment of bruxism [21,26].

Ultrasound can be used to evaluate masseter muscle volume, size, hypertrophy, and stiffness in patients with bruxism. In our study, ultrasound images before and after bruxism treatment were evaluated. Additionally, the ultrasonographic evaluation of the effect of some therapy methods on masseter muscle and blood flow in patients with bruxism, supports the potential benefits of physical interventions in managing bruxism symptoms [3]. These findings align with our study’s focus on predicting treatment outcomes and suggest that interventions can have positive effects on muscle parameters. With the findings of our study, the evaluation of the success of treatment methods for bruxism patients are not only based on subjective or quantitative concepts but can now also be confirmed with ultrasound images and radiomics. This will provide motivation for the patient and objective treatment prediction for the physician.

In this study, the muscles of bruxism patients before and after treatment were evaluated with radiomics. There are very few radiomic studies in bruxism in the literature yet. Although the publications using radiomic features are increasing day by day in the literature, there is no consensus on which of these radiomic features are reproducible [32]. The findings of the present study will provide valuable insights into the future opportunities of muscle ultrasound in various clinical contexts and for other populations. Images are transformed to look for correlations that may identify a useful radiographic phenotype before, during, or after treatment [33,34]. The most common use of ultrasound-based radiomic studies is to differentiate between benign and malignant tumors. There are many radiomic studies used for this purpose in structures such as breast and liver, especially in thyroid tissue [35,36,37]. There are many radiomic studies evaluating tumoral lesions in the literature, including a study by Lee et al., in which they aimed to distinguish triple-negative breast cancer from fibroadenoma using tissue analysis, and another by Zhang et al., in which they extracted radiomic features from 2064 pathologically confirmed B-mode and elastography images of thyroid nodules [38,39]. 

This review provides valuable insights into the present state and future opportunities of muscle ultrasound. It highlights the diverse applications of ultrasound imaging in assessing muscle morphology, architecture, and function [40]. Incorporating the knowledge and advancements discussed in this review can further enhance the interpretation and application of ultrasound-based machine learning techniques in our study. In the context of muscle segmentation, deep learning (DL) and atlas-based models have shown promise in medical image analysis. Studies like (Chen et al.) [41] and (Keser et al.) [42] utilized DL approaches for fast auto-segmentation of masticatory muscles on head and neck CT images and masseter muscle segmentation on ultrasonography, respectively. These approaches can contribute to improving the accuracy and reliability of muscle segmentation in our predictive model, thereby enhancing its overall performance.

Studies using ultrasound radiomic modeling in the field of health have been frequently seen in the literature recently. It is noteworthy that there are studies on cancer in general. A study on bruxism has not been conducted, to the best of our knowledge. Although not directly related to bruxism, the study by Saleh et al. on a DL localization method for measuring abdominal muscle dimensions in ultrasound images is relevant to our research [43]. Their DL approach for muscle segmentation in ultrasound images can enhance the accuracy and efficiency of our predictive model, as precise delineation of muscle boundaries is essential for accurate measurements and analysis. In a study by Qin et al. [44] in which they developed B-mode ultrasound radiomic models to identify the origin of primary tumors in metastatic liver disease with satisfactory AUC values reflected in the training and test sets, a total of 5936 features were extracted, and 40, 6, and 14 optimal features were sequentially identified for the development of radiomic models for groups 1, 2, and 3, respectively, with training set AUC values of 0.938, 0.974, and 0.768 and testing set AUC values of 0.767, 0.768, and 0.750. These values are close to our study. Qin et al. emphasized that B-mode ultrasound radiomic models can be effective additional tools to identify the source of hepatic metastatic lesions [44]. In the literature, there are also ultrasound-based radiomic studies examining the texture and content of the tissues, as well as the differentiation of benign malignant tumoral lesions. For example, there are many studies evaluating liver fibrosis [45,46,47].

Overall, our study highlights the potential of USG-based machine learning models in predicting the outcome of bruxism treatments. The identified radiomic features, along with clinical factors, can contribute to a comprehensive assessment of treatment outcomes. It is important to note that these findings are based on the specific dataset and methodology employed in this study, and further research is needed to validate and generalize these results. 

Nonetheless, the current study has some limitations. The major limitations of this study are its retrospective design and limited number of study population. Additionally, the study population was restricted to a single institution. With larger cohorts, those results might be stronger. The size of AI training datasets is critical for machine learning projects. To define the optimal amount of data you need, you have to consider a lot of factors, including project type, algorithm and model complexity, error margin, and input diversity. In this study, we aimed to apply the 10 times rule. In our study, there were nine clinical parameters plus USG, making a total of 10 parameters. Out of these 10 clinical parameters, our aim was to construct an ML algorithm to predict the outcome variables according to the 10-times rule. This rule means that the amount of input data (i.e., the number of examples) should be ten times more than the number of degrees of freedom a model has. Usually, degrees of freedom mean parameters in the dataset. Therefore, our AI training set should be at least 100 patients or images. Our clinical dataset consists of 102 patients. This is thought to be enough to construct an algorithm for prediction. Nevertheless, it should be stated that the more data, the better it is for ML studies.

## 5. Conclusions

This study proposed a machine learning model using SVM analysis on USG images for bruxism patients, capable of detecting changes in the masseter muscle. Additionally, this study demonstrated that combining specific US-based radiomics features with image variables can predict pain reduction in bruxism patients following treatment.

## Figures and Tables

**Table 1:** Radiomics features selected for quantifying heterogeneity differences.

Radiomics Group	Associated Filter	Radiomics Features
First-order statistics	None	Energy, total energy, entropy, minimum, 10 percentile, 90 percentile, maximum, mean, median, interquartile range, range, mean absolute deviation, robust mean absolute deviation, root mean square, standard deviation, skewness, kurtosis, variance
Shape	None	Volume, surface area, surface volume ratio, spherical disproportion, maximum 3D diameter, maximum 2d diameter column, maximum 2d diameter row, elongation
Texture features	GLCM	Autocorrelation, average intensity, cluster prominence, cluster shade, cluster tendency, contrast, difference average, difference entropy, difference variance, dissimilarity, entropy, sum average, sum entropy, sum variance, sum squares
Texture features	GLSZM	Large area emphasis, gray level non uniformity, size zone non uniformity, gray level variance, zone entropy, high gray level zone emphasis, small area high gray level emphasis, large area high gray level emphasis
Texture features	GLRLM	Gray level non uniformity, run length non uniformity, gray level variance, run entropy, high gray level run emphasis, short run high gray level emphasis, long run high level emphasis

Label: GLCM = Gray-Level Co-occurrence Matrix, GLSZM = Gray-Level Size Zone Matrix, GLRLM = Gray-Level Run Length Matrix.

**Table 2:** ROC analysis of the total training dataset by six classifiers.

Classifiers	AUC	95% CI	Sensitivity	Specificity
kNN	0.857	0.68–1.00	0.95	0.42
SVM	0.986	0.85–1.00	0.98	0.64
XGBoost	0.872	0.71–1.00	0.9	0.58
RF	0.84	0.74–0.96	0.98	0.14
LR	0.781	0.64–0.92	0.93	0.25
DT	0.772	0.64–0.91	0.92	0.29

kNN: k-Nearest Neighbor, SVM: Support Vector Machine, XGBoost: eXtreme Gradient Boosting, RF: Random Forest, LR: Logistic Regression, DT: Decision tree, AUC: Area Under Curve, CI: Confidence Interval.

**Table 3:** ROC analysis of the total test dataset by six classifiers.

Classifiers	AUC	95% CI	Sensitivity	Specificity
kNN	0.394	0.31–0.48	0.91	0.31
SVM	0.848	0.58–1.00	0.93	0.85
XGBoost	0.556	0.58–0.58	1.00	0.33
RF	0.679	0.68–0.68	1.00	0
LR	0.636	0.64–0.64	1.00	0
DT	0.611	0.27–0.97	0.91	0.33

kNN: k-Nearest Neighbor, SVM: Support Vector Machine, XGBoost: eXtreme Gradient Boosting, RF: Random Forest, LR: Logistic Regression, DT: Decision tree, AUC: Area Under Curve, CI: Confidence Interval.

**Table 4:** Evaluation for diagnostic performance by four indicators: precision, recall, F1-score, in the training set.

	Indicators	kNN	SVM	XGBoost	RF	LR	DT
1	Precision	0.84	0.91	0.88	0.81	0.80	0.83
	Recall	0.95	0.98	0.90	0.98	0.93	0.92
	F1-score	0.89	0.95	0.89	0.89	0.86	0.88
	Support	40	40	40	40	40	40
2	Precision	0.71	0.90	0.64	0.67	0.50	0.50
	Recall	0.42	0.64	0.58	0.14	0.25	0.29
	F1-score	0.53	0.75	0.61	0.24	0.33	0.36
	Support	12	12	12	14	53	12

KNN: k-Nearest Neighbor, SVM: Support Vector Machine, XGBoost: eXtreme Gradient Boosting, RF: Random Forest, LR: Logistic Regression, DT: Decision tree. “1” indicates normal. “2” bruxism patients. F1 = 2 * (precision * recall)/(precision + recall), support was defined as the number of samples of the true response that lies in each class of target values.

**Table 5:** Evaluation for diagnostic performance for the four indicators: precision, recall, F1-score, in the test set.

	Indicators	kNN	SVM	XGBoost	RF	LR	DT
1	Precision	0.77	0.85	0.81	0.79	0.78	0.81
	Recall	0.91	1.00	0.93	1.00	1.00	0.93
	F1-score	0.83	0.92	0.87	0.88	0.88	0.87
	Support	11	11	11	11	14	11
2	Precision	0.00	1.00	0.50	0.00	0.00	0.50
	Recall	0.00	0.33	0.25	0.00	0.00	0.25
	F1-score	0.00	0.50	0.33	0.00	0.00	0.33
	Support	3	3	3	3	4	2
Cumulative	Precision	0.77	0.85	0.79	0.79	0.79	0.81
	Recall	0.91	1.00	0.95	0.95	0.93	0.91
	F1-score	0.83	0.91	0.84	0.83	0.88	0.87
	Support	11	11	11	11	11	11

kNN: k-Nearest Neighbor, SVM: Support Vector Machine, XGBoost: eXtreme Gradient Boosting, RF: Random Forest, LR: Logistic Regression, DT: Decision tree. “1” indicates normal. “2” bruxism patients. F1 = 2 * (precision * recall)/(precision + recall), support was defined as the number of samples of the true response that lies in each class of target values. Cumulative represents the discrimination between bruxism and control patients according to evaluation based on the test set.

**Table 6:** Support Vector Machine regression coefficients of selected features in the clinical, radiomic, and combined model in the prediction of pain reduction in the total patient cohort (*n* = 78 bruxism patients).

	Selected Features	SVMR Coefficients	*p*-Value
Patient reduction	**Clinical model**		
	Age	−0.582	
	Gender	0.510	
	Maximum mouth opening	0.760	
	Pain (Vas score)	0.620	
	Pain on palpation	−0.120	0.016 †
	Treatment type (KTMT)	0.510	0.02 †
	Treatment type (MT)	0.510	0.03 †
	Treatment type (BT-A)	−0.059	
	Sleep quality index	0.448	
	**Radiomic model**		
	Ibp-2D_firstorder_variance	−0.880	0.04 †
	Ibp-2D_firstorder_10percentile	−0.330	0.02 †
	Exponential_glszm_ZoneVariance	0.442	
	Wavelet_LLH_glszm_SmalldepedneceHighGrayLevelEmphasis	0.650	
	Wavelet_HLH_glszm_SmallAreaHighGrayLevelEmphasis	0.152	0.03 †
	Wavelet-LLL_firstorder_variance	0.065	0.03 †
		0.442	
	**Combined model**		
	Age *	−0.109	
	Gender *	0.335	
	Treatment type (KTMT) *	0.079	0.021 †
	Treatment type (MT) *	0.218	0.02 †
	Treatment type (BT-A) *	0.046	0.018 †
	Pain on palpation *	−0.110	0.02 †
	Ibp-2D_firstorder_variance *	0.280	
	Ibp-2D_firstorder_10percentile *	0.228	
	Exponential_glszm_ZoneVariance *	0.091	0.03 †
	Wavelet_LLH_glszm_SmalldepedneceHighGrayLevelEmphasis *	0.333	0.02 †
	Wavelet_HLH_glszm_SmallAreaHighGrayLevelEmphasis *	0.541	0.02 †
	Wavelet-LLL_firstorder_variance *	0.440	

Negative coefficients indicate a higher likelihood of pain. Features included in both the radiomic and combined model are summarized with an asterisk. Associations between each radiomic feature and outcomes were calculated using a Mann–Whitney-U test (*p* < 0.05 were considered significant (†)). * indicates the clinical and radiomic features that was included to combined model.

## Data Availability

The datasets used and/or analyzed during the current study are available from the corresponding author on reasonable request.
